# The Origin and Evolution of G Protein-Coupled Receptor Kinases

**DOI:** 10.1371/journal.pone.0033806

**Published:** 2012-03-19

**Authors:** Arcady Mushegian, Vsevolod V. Gurevich, Eugenia V. Gurevich

**Affiliations:** 1 Stowers Institute for Medical Research, Kansas City, Missouri, United States of America; 2 Department of Microbiology, Kansas University Medical Center, Kansas City, Kansas, United States of America; 3 Department of Pharmacology, Vanderbilt University, Nashville, Tennessee, United States of America; Medical School of Hannover, United States of America

## Abstract

G protein-coupled receptor (GPCR) kinases (GRKs) play key role in homologous desensitization of GPCRs. GRKs phosphorylate activated receptors, promoting high affinity binding of arrestins, which precludes G protein coupling. Direct binding to active GPCRs activates GRKs, so that they selectively phosphorylate only the activated form of the receptor regardless of the accessibility of the substrate peptides within it and their Ser/Thr-containing sequence. Mammalian GRKs were classified into three main lineages, but earlier GRK evolution has not been studied. Here we show that GRKs emerged at the early stages of eukaryotic evolution via an insertion of a kinase similar to ribosomal protein S6 kinase into a loop in RGS domain. GRKs in Metazoa fall into two clades, one including GRK2 and GRK3, and the other consisting of all remaining GRKs, split into GRK1-GRK7 lineage and GRK4-GRK5-GRK6 lineage in vertebrates. One representative of each of the two ancient clades is found as early as placozoan *Trichoplax adhaerens*. Several protists, two oomycetes and unicellular brown algae have one GRK-like protein, suggesting that the insertion of a kinase domain into the RGS domain preceded the origin of Metazoa. The two GRK families acquired distinct structural units in the N- and C-termini responsible for membrane recruitment and receptor association. Thus, GRKs apparently emerged before animals and rapidly expanded in true Metazoa, most likely due to the need for rapid signalling adjustments in fast-moving animals.

## Introduction

The discovery of activation-dependent rhodopsin phosphorylation almost 40 years ago [1], [2] was soon followed by the description of “opsin kinase” (modern name GRK1) that selectively phosphorylates light-activated rhodopsin [3]. Rhodopsin phosphorylation was shown to be necessary for its rapid deactivation, which led to the idea that similar mechanism may regulate the signalling by hormone receptors [4]. This hypothesis was proved for β2-adrenergic receptors [5], [6] and many other GPCRs (reviewed in [7]). Interestingly, sequence similarity between β2-adrenergic receptor (β2AR) and rhodopsin, revealing the existence of the family of G protein-coupled receptors (GPCRs), was not discovered until later [8]. The demonstration that rhodopsin phosphorylation greatly increases its affinity for another protein, arrestin (at the time called 48-kDa protein), which actually blocks further signaling [9], provided the first clue to the biological role of receptor phosphorylation. Beta-adrenergic receptor kinase (βARK; modern name GRK2) that specifically phosphorylates active β2AR was identified next [10]. Surprisingly, it was also able to phosphorylate rhodopsin in light-dependent manner [11]. Finally, a homolog of arrestin was shown to participate in the desensitization of the β2AR [12]. Collectively, these studies established the paradigm of two-step receptor inactivation, which is valid for the great majority of GPCRs [7], [13], [14]. The sequence of the first member of GRK family, βARK, suggested that it likely belongs to a distinct branch of eukaryotic Ser-Thr protein kinases [15]. It is currently classified as GRK superfamily within the AGC kinase group [16]. This superfamily was expanded by cloning of βARK2 (GRK3) [17], GRK4 [18], GRK5 [19], and GRK6 [20], followed by cone-specific GRK7 [21].

Based on the relative extent of sequence similarity, vertebrate GRKs have been traditionally divided into three subfamilies: GRK1, comprising GRK1 (rhodopsin kinase) and GRK7 (cone opsin kinase); GRK2, comprising GRK2 and 3; and GRK4 comprising GRK4, 5, and 6. All GRKs are multidomain proteins (see Fig. 1 in [22]) consisting of ∼30-residue N-terminal region specific for this family, followed by the Regulator of G protein Signaling (RGS) homology domain (RH) [23], and a Ser-Thr protein kinase domain (KD) with high similarity to other AGC protein kinases, such as PKA, PKB, and PKC. This ∼500–520 amino acids long domain assembly is shared by all GRKs. The C-termini of GRKs contain additional structural elements responsible for their membrane targeting: GRK1 and 7 carry short C-terminal prenylation sequences, GRK2 and 3 contain pleckstrin homology (PH) domain interacting with G protein βγ-subunits [24], GRKs 4 and 6 carry palmitoylation sites, whereas GRK5 has positively charged lipid-binding element. This domain composition correlates with the degree of sequence similarity in the shared domains, supporting the same three subfamilies of GRKs in vertebrates. Crystal structures of one representative from each subfamily were solved: GRK2 alone [25] and in complex with G protein βγ-subunit [26] or with both Gq α-subunit and βγ-subunit [25], GRK6 [27], and GRK1 [28]. The structures suggest that RH+KD core of GRKs appeared as the result of the insertion of KD into α9-α10 loop of the ancestral RH domain [26], [27], and that different family members subsequently acquired various additional structural elements. One feature distinguishing GRKs from other AGC kinases is that the two lobes of the KD are in the “open” orientation and “nucleotide gate” peptide is disordered regardless of the presence of the ATP analogue or G protein subunits [27]. This indicates that the kinase requires an induced rearrangement to become active, which is apparently provided by the binding to active GRCRs, consistent with biochemical data [29], [30]. The three-dimensional structures reveal extensive flat surface with abundant positive charges that likely faces the membrane. This conclusion is supported by the finding that lipid anchors of bound G protein subunits also localize to the same surface [26], [31]. GRK N-terminus and several receptor-facing residues in the kinase domain appear to mediate allosteric activation of GRKs by the active GPCRs [32], [33], [34]. The binding of other proteins, such as GRK1 regulator recoverin, to the N-terminus inhibits receptor-dependent kinase activation [35].

Here we survey the presence of GRK-related proteins, defined by the RH+KD domain architecture, and outline the evolutionary history of GRK family. The evidence suggests that the common ancestor of all Metazoa most likely had two GRK-like kinases, one giving rise to the GRK2/3 lineage and the other undergoing a later split into the GRK1/7 and GRK 4/5/6 lineages.

## Results

### The origin and evolution of GRKs

To extract GRK sequences, the psi-blast search with the kinase domain of *Enteroctopus dofleini* (giant pacific octopus) GRK (gi 4519169) was performed, as described in Methods. This sequence was selected to avoid biasing the search towards chordate GRKs. After extracting all known and predicted GRKs from the model organisms, we manually removed sequences with incomplete KD, shorter alternatively spliced variants, and duplicate sequences (Table S1). This procedure identified total 151 GRKs in vertebrates and invertebrates, including cephalopod mollusks and model organisms with completely sequenced genomes, such as primitive chordates *B. floridae* and *C. intestinalis*, fruit fly and other insects, nematode *C. elegans*, sea anemone *N. vectensis*, and primitive metazoan *T. adherens*. Interestingly, we also detected the RH+KD fusions in non-metazoan species, including the opisthokont *Monosiga brevicolis*, thought to be the unicellular eukaryote closest to Metazoa [36]; another opisthokont *Capsaspora owczarzaki* (ATCC 30864), a unicellular amoeboid parasite of tropical snails, that has recently emerged as another candidate sister clade of Metazoa [37]; late blight oomycete *Phytophotra infestans*; white rust oomycete *Albugo laibachii* Nc14; and brown alga *Ectocarpus siliculosus*. All sequenced genomes of invertebrates and of a cephalochordate have two GRK-like genes, whereas all unicellular organisms have only one.

**Figure 1:**
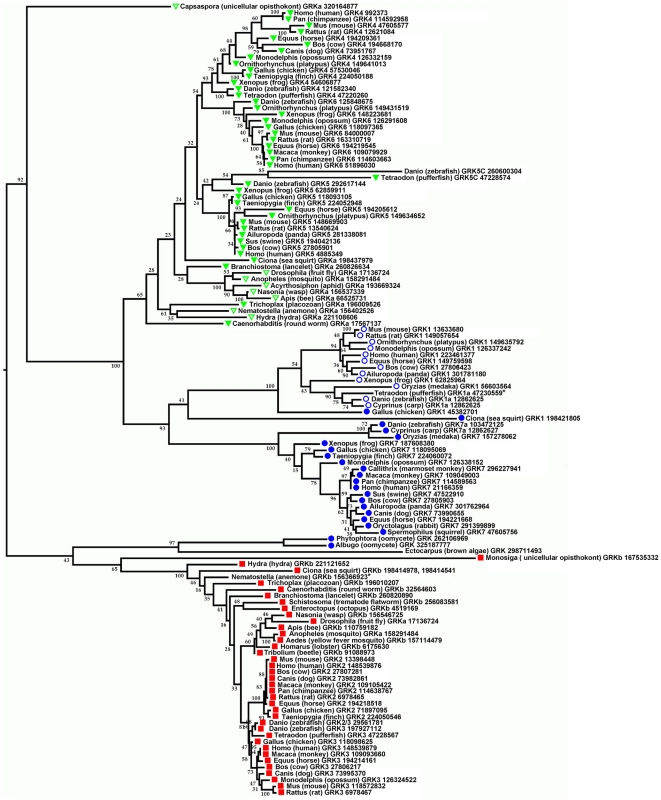
Phylogeny of the kinase domains of GRK proteins. Bootstrap support (in percent) of each partition is indicated by the numbers at the internal branches. The NCBI GI numbers are given for each sequence. The symbols next to the species name indicate the type of the C-terminal structure found in each GRK sequence: red squares indicate C-terminal PH domains; blue circles indicate C-terminal prenylation motifs: geranylgeranylation (solid circle) or farnesylation (open circle); and green triangles indicate either predicted canonical (solid triangle) or putative modified (open triangle) C-terminal amphipathic helix. Note that the C-terminal motives generally group with the GRK families. Thus, GRKb/2/3 family possesses C-terminal PH domain in most species including unicellular opisthokont Monosiga; the GRKa/4/5/6 family - the canonical of modified predicted amphipathic helix (including unicellular opisthokont Capsaspora), and GRK1/7 family - prenylation motives. However, early GRKs from unicellular oomycetes Phytophthora and Albugo, although loosely grouping with the GRKb/2/3 family, have prenylation motives at the C-terminus, although certain elements of the PH domain can also be detected. The GRK from brown algae Ectocarpus lacks any recognisable C-terminal motive and so does GRK5C from Danio. Species abbreviations are as follows: Acyrthosiphon, *Acyrthosiphon pisum* (pea aphid); Aedes, *Aedes aegypti* (yellow fever mosquito); Ailuropoda, *Ailuropoda melanoleuca* (giant panda); Anopheles, *Anopheles gambiae* (mosquito); Albugo, *Albugo laibachii* (oomycete); Apis, *Apis mellifera* (honey bee); Branchiostoma, *Branchiostoma floridae* (Florida lancelet; cephalochordate); Bos, *Bos taurus* (cow); Caenorhabditis, *Caenorhabditis elegans* (round worm; nematode); Callithrix, *Callithrix jacchus* (white-tuft-ear marmoset); Canis, *Canis lupus familiaris* (dog); Caspospora, *Caspospora owczazaki* (unicellular opisthokont); Ciona, *Ciona intestinalis* (sea squirt); Cyprinus, *Cyprinus carpio* (common carp); Drosophila, *Drosophila melanogaster* (fruit fly; insect); Danio, *Danio rerio* (zebrafish; teleost fish), Ectocarpus, *Ectocarpus siliculosus* (brown alga); Enteroctopus, *Enteroctopus dofleini* (giant octopus); Equus, *Equus caballus* (horse); Gallus, *Gallus gallus* (chicken); Homarus, *Homarus americanus* (American lobster); Homo, *Homo* sapience (human); Hydra, *Hydra magnipapillata* (hydra); Monodelphis, *Monodelphis domestica* (gray short-tailed opossum); Monosiga, *Monosiga brevicolis* (unicellular opisthokont); Mus, *Mus* musculus (mouse); Macaca, *Macaca mulatta* (monkey); Nasonia, *Nasonia vitripennis* (Jewel wasp); Nematostella, *Nematostella vectensis* (starlet sea anemone; cnidarian); Ornithorhynchus, *Ornithorhynchus anatinus* (platypus); Oryctolagus, *Oryctolagus cuniculus* (rabbit); Oryzias, *Oryzias latipes* (Japanese medaka fish); Pan, *Pan troglodites* (chimpanzee); Phytophtora, *Phytophtora infestans* (oomycete); Rattus, *Rattus norvegicus* (rat); Schistosoma, *Schistosoma mansoni* (trematode flatworm); Spermophilus, *Spermophilus tridecemlineatus* (thirteen-lined ground squirrel; Sus, *Sus scrofa* (pig); Taeniopygia, *Taeniopygia guttata* (zebra finch); Trichoplax, *Trichoplax adhaerens* (placozoan); Tetraodon, *Tetraodon nigroviridis* (green pufferfish); Tribolium, *Tribolium castaneum* (beetle); Xenopus, *Xenopus laevis* (African clawed frog). * - the sequence is truncated at the C terminus.

We have extended these observations by building phylogenetic tree (Fig. 1) based on the aligned KD domains shared by the GRK homologues. The presence of RH domain was obligatory for the inclusion of the sequence in the analysis (to consider the sequence to be that of a GRK-like protein) but the RH domain itself was not used in the analysis, because it was incomplete in several of the predicted proteins., The Maximum-Likelihood tree clearly shows two early splits that produced three statistically well-supported groups. The same well-supported splits were observed in the Bayesian trees (data not shown). The first split partitioned the ancestor of the GRK2/GRK3 group and the rest of the GRKs, and the second split separated GRK1/7 and GRK4/5/6 in vertebrates (Fig. 1). Whenever a complete genome encodes two full-length GRKs, one of the two protein products belongs to the GRK2/3 clade and the other to the GRK(1/7)4/5/6 clade.

All proteins in the GRK2/3 clade, from primitive metazoans to vertebrates, have an additional shared feature, the C-terminal PH domain. In *C. elegans*, there are two GRK genes, one of which, called *grk-2*, encodes a product with 66% identity with human GRK3 and similar domain structure [38]. In *Drosophila*, one of the two GRK isoforms, photoreceptor-enriched GRK1, is highly similar to the mammalian GRK2 and GRK3 and has the same domain structure [39]. In the same clade are GRKs from cephalopod mollusks, which express exclusively in photoreceptors [40] and have been annotated as rhodopsin kinases because of that, even though they are not in the same clade as human GRK1. In Fig. 1, we call the members of this clade present in invertebrate model organisms (and a cephalochordate *B. floridae*) GRKb, to avoid the impression of their specific association with mammalian GRK1 branch (or with GRK2 as opposed to GRK3, a distinction not relevant in invertebrates). Interestingly, the sole known GRK-like protein in unicellular *Monosiga* is placed into this group on the basis of similarity in KD with moderately significant bootstrap support of 65%, and it also has the C-terminal PH domain.

The other clade is split into two subgroups, vertebrate-specific clade consisting of rhodopsin (GRK1) and cone opsin (GRK7) kinases, and the other clade comprising mammalian GRK4, 5 and 6 and their single-copy orthologs in lancelet and invertebrates. These proteins are more diverse than the members of the GRK2/3 clade. In *C. elegans*, this clade is represented by grk-1 gene product and in *Drosophila* by ubiquitous GRK2; in Fig. 1, we relabeled them as GRKa, in order to emphasize that they do not belong to the clades specified by mammalian GRK1 or GRK2. Several other members of this group from invertebrates, e.g., from mosquito and from cnidaria, were annotated as GRK4, but we suggest that they should be labeled GRKa, as they are orthologous to the whole GRK4/5/6 group in vertebrates, and do not show a specific connection to GRK4 to the exclusion of GRK5 and GRK6. The same is true for the sole member of this clade in the cephalochordata *B. floridae*. The sole known GRK homolog from opisthokont *C. owczarzaki* is also robustly placed into this clade (bootstrap 92%).

Examination of the phylogenetic tree provides several other notable observations. First, a hemichordate *C. intestinalis* appears to represent an intermediate step in the evolution of the two clades. It has three GRKs, one of which is a basal member of GRK4/5/6 clade (renamed GRKa for this reason), another (GRK1) is a basal member of the “visual” branch in the same clade (Fig. 1), and the third (renamed GRKb) is the member of the GRK2/3 clade, where it mingles with the invertebrate GRKb homologs. Second, whereas both GRKa and GRKb of primitive metazoans *N. vectensis* and *T. adhaerens* are correctly resolved as the nearest neighbours within the GRK4/5/6 and GRK2/3 clade, respectively, the placement of the other orthologs is not always consistent with the species' phylogenetics tree: for example, both GRKa and GRKb from *C. elegans* are deep branches within respective clades, failing to show specific affinity with arthropods that is implied by the Ecdysozoa hypothesis [41].

Third, the set of GRKs in zebrafish includes pairs of closely related paralogs of GRK3, which may be consistent with the apparent whole-genome duplication in teleost fish lineage [42]; there are no paralogs of GRKs 4 or 6, however, and no ortholog of GRK2, suggesting post-duplication gene losses. Zebrafish genome contains two GRK5 paralogs. One protein (designated GRK5C) is particularly rapidly evolving and is robustly placed, together with the pufferfish (*Tertaodon nigrovidis*) ortolog into a separate branch (bootstrap 85%). It also lacks any recognizable C-terminal motifs typical of the GRKa/4/5/6 family, although the pufferfish ortholog has a characteristic amphipathic helix. In the zebrafish genome, a pair of sequences has been annotated as GRK1 (designated GRK1a and GRK1b) and GRK7 (GRK7a and b) (Table S1; not shown on the tree to avoid overcrowding). The sequences are closely related and may have resulted form the whole-genome duplication. Genomes of other teleost fishes (carp, pufferfish, Japanese medaka) appear to contain paralogs of GRK1 (Table S1).

Fourth, in addition to the three main branches in the tree, the GRK-like putative proteins from oomycetes and an alga form their own clade, apparently preceding the split into GRKa/1/7/4/5/6 and GRKb/2/3 in Metazoa and their unicellular relatives.

### Organization of GRK genes

**Figure 2:**
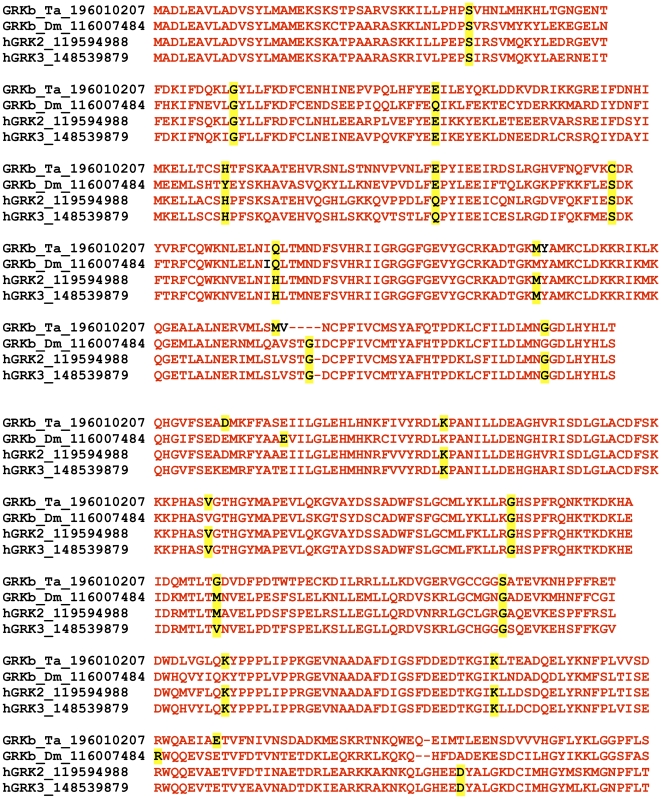
Intron positions in the GRK2/GRK3 clade of G protein-coupled receptor kinases are highly conserved in metazoan evolution. Human GRK2 and GRK3 are aligned to their orthologs from an insect (*Drosophila melanogaster*, Dm) and a primitive metazoan (*Trichoplax adhaerens*, Ta). The amino acids with codons either interrupted or immediately followed by an intron in the genome sequence are highlighted by yellow shading. Ten positions are conserved in all four genes, seven additional positions are conserved between human genes and at least one of the invertebrate orthologs, and only five non-conserved positions were identified.

The ancient origin of the characteristic RH+KD domain organization of GRKs is also supported by the analysis of the intron positions in the genes of GRK2/3 clade, the vast majority of which are found in the orthologous codons in higher and lower metazoans, suggesting the shared exon-intron structure of the ancestral GRK2/3 gene (Fig. 2). The exon positions were not as conserved in the other branch of GRK family (not shown).

### Evolution of N-terminal receptor binding elements

**Figure 3:**
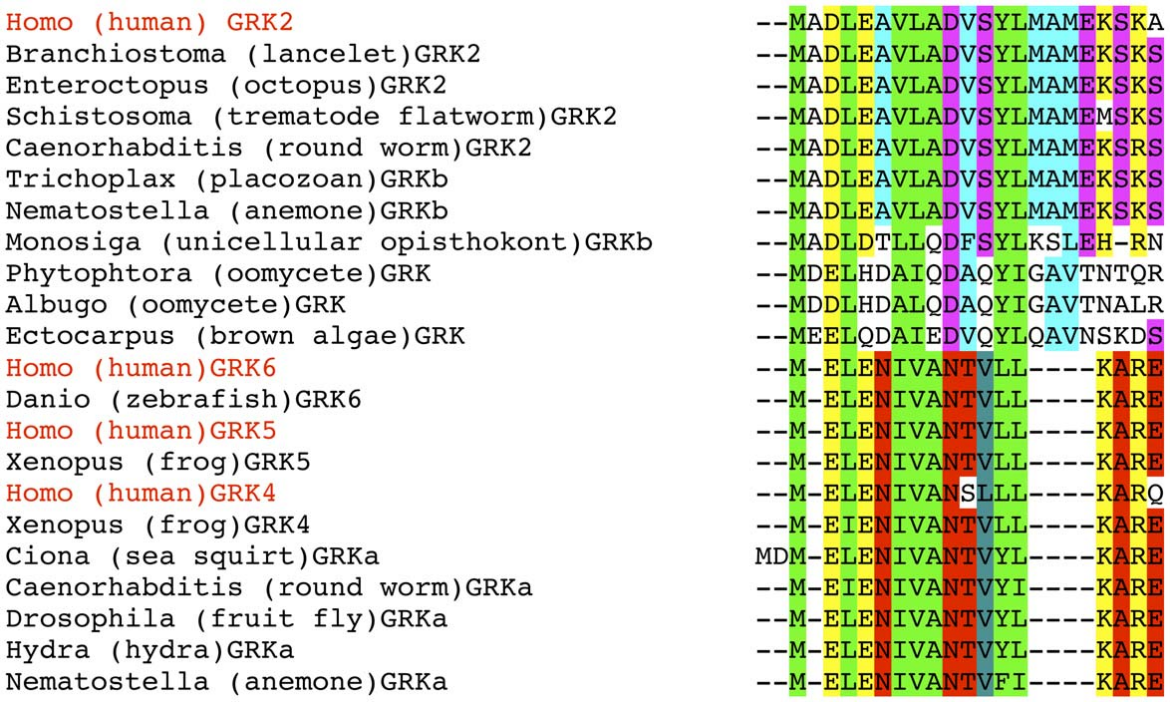
Conservation of GRK-specific N-terminal helix implicated in GPCR binding. Hydrophobic residues conserved in both GRKb/2/3 and GRKa/4/5/6 clades are highlighted in green; hydrophobic residues conserved within the GRKb/2/3 are highlighted in light blue, within the GRKa/4/5/6 clade - in dark blue-green. Other residues conserved in both clades are highlighted in yellow; residues conserved within the GRKb/2/3 are highlighted in pink, within the GRKa/4/5/6 clade - in red. In all cases conservative substitutions are also highlighted. Note that all kinases in the GRKa/4/5/6 are missing four residues that are highly conserved in the GRKb/2/3 clade. This alignment suggests that GRKs from both oomycetes and brown alga group with GRKb/2/3 clade. Species abbreviations are as in Fig. 1.

The N-terminus of GRKs contains sequence specific for this family and critical for the kinase binding to active GPCRs [32], [34], [43]. The N-terminal sequence of GRK2 clade shows very high conservation of multiple residues across species (Fig. 3), including sea anemone *N.vectensis*, placozoan *T. adherens*, and non-metazoan *M. brevicolis*. Multiple residues and the overall helical structure are conserved in GRKs from monocellular oomycetes *Phytophtora*, *Albugo*, and brown alga *Ectocarpus*, supporting tentative placement of predicted GRKs from these organisms into GRKb/2/3 clade based on the kinase domain alignment and phylogenetic tree analysis (Fig. 1). In both clades the distribution of hydrophobic and charged side chains within the first 13 residues is compatible with the formation of amphipathic α-helix.

**Figure 4:**
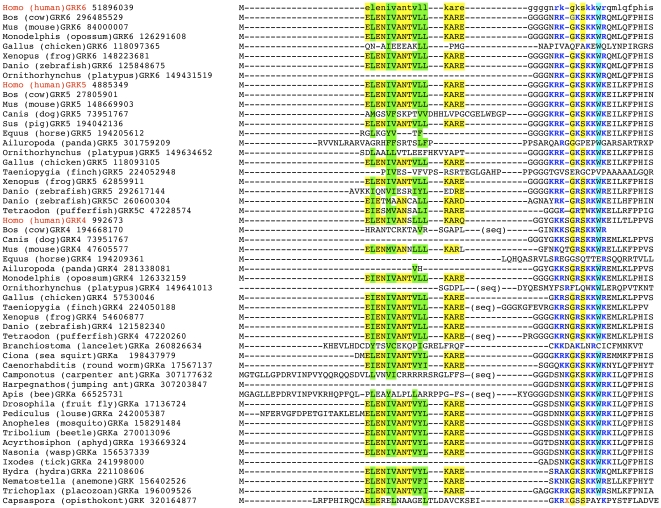
Structural features of the N-termini of the GRKa/4/5/6 group. The N-terminal α-helix present in all GRKs (Fig. 3), in the GRKa/4/5/6 clade is followed by several glycines that break secondary structure and highly conserved sequence containing (in this order) 1–3 positively charged residues, glycine, a positively charged residue, serine, two positive charged residues, tryptophan, and 1–2 positively charged residues. The absence of this sequence and/or N-terminal helix, which is remarkably conserved from *Trichoplax* to human, in some predicted GRK sequences (platypus GRK6, finch GRK5, dog, horse, panda, and platypus GRK4, and GRKa from tick and jumping ant) likely indicates that these parts of corresponding genes were missed or erroneously annotated. GRKa from opisthokont *Capsaspora owczarzaki* appears to have rudimentary versions of the helix and positive patch. Highlighting: the α-helix, conserved hydrophobic residues in green, other conserved residues in yellow; the positive patch: positively charged residues are shown in bold blue, conserved glycine, and serine are highlighted in yellow, conserved tryptophan in blue. (seq) indicates a sequences interposing between the receptor-binding N-terminal and α-helix a stretch of glycines followed by a conserved hydrophobic motive. These sequences were removed to avoid long gaps. Considering that the N-terminal sequences in human GRK5/6 and Trichoplax GRKa are nearly identical, it appears likely that these sequences simply resulted from mispredictions. Apparent insert in *C. owczarzaki* GRK is shown in red as X, which stands for the sequence LFLTHFC. Species abbreviations are as in Fig. 1.

The N-terminal sequence of most Metazoan GRKs in the GRKa/4/5/6 clade contains another characteristic element. Predicted α-helix is followed by several structure-breaking glycines, followed by XX**G**X**S**XX**W**X, where X is a positively charged residue, lysine or arginine (Fig. 4). In the structure of the most closed conformation of GRK6 this sequence creates an intensely basic, relatively flat surface immediately adjacent to the protruding N-terminal α-helix that was predicted to participate in GRK binding to the membrane [34]. This highly basic motif is remarkably conserved throughout the animal kingdom. In fact, the sequences of the human GRK5 and in GRKa from *T. adherens* in this region are identical. In GRKa from non-metazoan opisthokont *C. owczarzaki*, there is an insert between starting basic residues (KR) and GXS core, and two out of three typical basic residues are substituted by non-charged amino acids.

### Evolution of C-terminal sequences in the GRKa/4/5/6 subfamily

GRKs are soluble, whereas their best-characterized substrates, GPCRs, are integral membrane proteins. GRK membrane localization is mediated largely, although not exclusively, by special sequence motifs in the C-termini, and GRK proteins have evolved different mechanisms for recruitment or attachment to the plasma membrane [reviewed in [22]].

**Figure 5:**
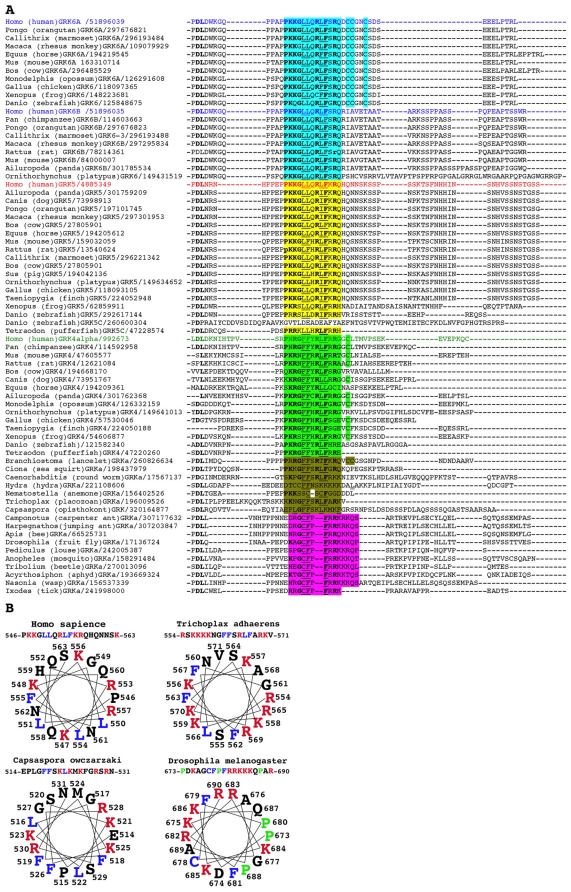
Characteristic features of the C-termini of GRK(1/7)4/5/6 clade. **A.** The C-terminal sequences of the kinases of GRKa/(1/7)4/5/6 clade were aligned. The most conserved parts of the putative amphipathic helix and putative palmitoylation sites are highlighted in blue (GRK5), yellow (GRK6), green (GRK4), light brown (invertebrate GRKa), or magenta (insect GRKa). Cysteines (potential palmitoylation sites) downstream of the helix are highlighted in the same color as the helix in corresponding branch. Residues conserved in the entire clade (DL at the beginning of the alignment) or in the helix in individual branches are shown in bold. Characteristic pairs of hydrophobic residues within the helix are underlined. Species abbreviations are as in Fig. 1. **B.** The structure of the amphipathic helix in the C-terminus of human GRK5 and putative helical structures of homologous elements in GRKa from *T. adherens*, *C. owczarzaki*, and *D. melanogaster*. Hydrophobic residues are shown in blue, positively charged in red. Note that the structure of this element in GRKa of *Drosophila* is less likely to be helical due to the presence of three pralines (shown in green), although this is not the case for all insect GRKas. In any case, a stretch of positive charges in this GRK can also serve as the membrane anchor. The sequence of this element in each species, with the numbers of the first and last residue at the beginning and the end, respectively, are shown above the helical diagrams.

In the GRK4 subfamily, the main membrane targeting mechanism is via the binding to phospholipids. An amphipathic helix has been identified in the C-terminus of mammalian GRKs 4, 5 and 6, which contains hydrophobic residues on one side surrounded by a number of positively charged residues [44], [45]. We aligned the C-termini of all GRKs belonging to the GRK4/5/6 subfamily (Fig. 5A). The sequences of the amphipathic helices in GRKs 4, 5, and 6 (both A and B isoforms) are highly conserved in vertebrates. Cysteines that upon palmitoylation promote membrane localization of GRK6A [45], [46] and GRK4 [47] are also mostly conserved in vertebrates. The only exception is the fast-evolving GRK5 variant found in zebrafish (*D. rerio*) that did not have any recognizable C-terminal motif. *C. elegans* and *C. intestinalis* have two characteristic pairs of hydrophobic residues (**FF**XX**LF** motive found in GRK4) surrounded by many conserved positively charged residues. In *B. floridae*, leucine in the second pair is substituted by threonine, but there are two putative palmitoylation residues as in GRK6A, which are absent in *C. elegans* or *C. intestinalis*. The insect species evolved a unique C-terminal sequence consisting of a hydrophobic pair CF (not found in other species except cnidarian *N. vectensis*) followed by PF and a stretch of positively charged residues. Although three hydrophobic residues are present, the amphipathic helix is less likely due to the presence of three prolines; overabundance of positively charged residues suggests that in insect GRKs this element is more likely to be a membrane-binding positive patch (Fig. 5B). These structures are likely able to promote the membrane attachment, with hydrophobic residues directly inserting into the lipid bilayer and positively charged residues binding to the head groups of anionic phospholipids. There is little similarity in the C-terminal sequence between the *C. elegans* and insect GRKs, again giving no support to special closeness of nematodes to arthropods implied by Ecdysozoa hypothesis [41].

Interestingly, a well-defined amphipathic helix is present in the GRK of *T. adhaerens* (Fig. 5B), a placozoan considered to be the basal eumetazoan preceding the divergence of cnidarians and bilaterians [48]. The GRK from a cnidarian *H. magnipapillata* shows an imperfect amphipathic helix, whereas a single GRK from another cnidarian *N. vectensis* lacks hydrophobic helical core (there is only one pair, CF, the same as in insects) as well as positively charges residues, which may be due to a secondary loss. The GRK of the opisthokont *C. owczarzaki*, the earliest known GRK of the GRK4/5/6 subfamily, is missing phenylalanine in the second hydrophobic pair. However, close by there are two more hydrophobic residues, leucine and phenylalanine, able to form a stretch of hydrophobic residues on one side of the helix (Fig. 5B). The data suggest that the lipid binding structure such as an amphipathic helix was acquired very early in the C-terminus of the ancestor of GRKs 4/5/6 to enable the kinase to associate with the plasma membrane.

### Evolution of the PH domain

**Figure 6:**
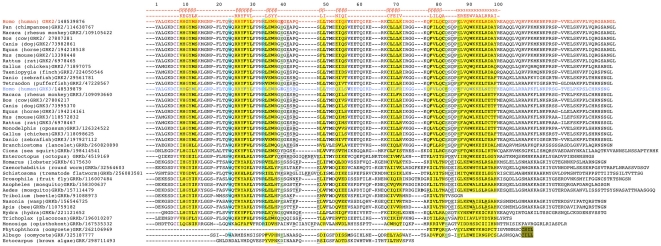
Plekstrin homology domain in the C-termini of GRKb/2/3 clade. The C-terminal sequences of the kinases of GRKb/2/3 clade and three GRKs from single-cell non-metazoan species *Phytophtora infestans* (apicomplexa), *Albugo laibachii* (oomycete), and *Ectocarpus siliculosus* (brown alga). PH domain residue numbering is based on the human GRK2. Conserved elements of the secondary structure and corresponding consensus sequences are shown above the alignment. Conserved core hydrophobic residues are shown in bold; conserved hydrophobic residues outside of the core are shown in bold blue; putative elements of the secondary structure are highlighted in yellow; conserved in GRK family W and R are highlighted in blue; conserved negative charges are shown in green bold underlined. Human GRK2 and GRK3 sequences are shown in red and light blue, respectively. Note incomplete set of elements of the putative PH domain in GRKs from single-cell pre-metazoan species. Species abbreviations are as in Fig. 1.

The members of the GRK2/3 subfamily are equipped with the C-terminal PH domain that mediates their membrane recruitment via binding to G protein Gβγ subunits [49], [50], [51], [52]. The PH domain is highly diverged at the sequence level, but its three-dimensional structure is conserved in different proteins (reviewed in [53]). The basic structure consists of a pair of β-sheets with four and three antiparallel strands and the C-terminal α-helix [53]. We found good sequence conservation in the PH domain in vertebrate and invertebrate GRKs (Fig. 6). Among Craniata species, both β-strands and inter-strand insertions are conserved. Invertebrates display more variability in β-strands and in the length and sequence of insertions. However, core hydrophobic residues remain conserved in all insects, two mollusc species, and one chidarian (*H. magnipapillata*). That includes core residues (PH domain-specific numbering, Fig. 6) M13, L31, L36, W38, L49, M51, I54, L68, L70, F78, L80, W91, and L95. A core F29 strictly conserved in Craniata is often substituted in invertebrates with neutral amino acids. The core C82 is strictly conserved in vertebrates but not in other species. Two non-core hydrophobic residues, V57 and C67, are also highly conserved. A *T. adhaerens* GRKb shows conservation of core hydrophobic residues and substantial sequence similarity with the invertebrate GRKs, particularly in β-strands (Fig. 6). Most variability is seen in the α-helix that is missing characteristic hydrophobic and charged residues. Similarly, in a single GRK found in a choanoflagellata *M. brevicolis*, a substantial homology, particularly in more upstream β-strands can be perceived, with the conservation of core Gly and most α-helix hydrophobic residues. This suggests that an overall fold of the domain is preserved, although the structure of the C-terminal α-helix is the least conserved. Interestingly, the W91 within the α-helical loop that is almost invariable in PH domains from different mammalian proteins [53], [54], [55] is conserved in GRKs in all species except *T. adhaerens* and *M. brevicolis*.

An important feature of PH domain is a conserved distribution of charged residues forming positively charged surface on one side and negatively charged on the other [53]. In PH domains of many proteins, the insertion between β_C_ and β_D_ is populated almost entirely by charged residues [54]. In mammalian GRKs glutamates E41 and E43 are present. Both are conserved in vertebrates and *B. floridae*. E41 is conserved in most invertebrates that also possess one or more additional charged residues in this loop (Fig. 6). Another position enriched with charges is the insertion between β_G_ and α-helix, where charged residues favour helix formation. In vertebrate GRKs, D83 (or E83 in GRK3s), D85, and E87 (the latter is the first amino acid in the α-helix) are highly conserved. In GRK from *B. floridae* all three residues are present, whereas in *C. intestinalis* D85 is substituted by glutamine. Invertebrates are missing this particular triplet, but they have their own arrangement of negatively charged amino acids consisting of D84-E85 pair. Cnidarian *H. magnipapillata* retains the same arrangement as in vertebrates, D83-D85-E87, whereas *T. adhaerens* and *M. brevicolis* only have E83 and no other charges.

The earliest GRKs from oomycetes and an alga, which form their own clade loosely associated with the GRKb/2/3 subfamily on the basis of aligned KD domains, lack recognizable lipid-binding motifs found in the GRKa/4/5/6 subfamily, nor do they possess a *bona fide* PH domain (Fig. 6). However, certain similarities are notable. For example, terminal α-helix of the PH domain is discernible in GRKs from *P. infestans* and *A. laibachii*. The starting sequence EYLEW is found in a cnidarian *H. magnipapillata* as EYNEW, and EW motif is present in all arthropods. Interestingly, GRKs in *P. infestans* and *A. laibachii* both possess C-terminal geranylgeranylation motives (CSIL and CILL, respectively) normally found in cone kinases (GRK7s) that would allow these GRK to semi-permanently associate with the plasma membrane.

## Discussion

Future phylogenetics analysis, including more extensive taxonomic sampling of unicellular relatives of Metazoa, invertebrates and primitive vertebrates will provide a more detailed understanding of evolutionary events that shaped the GRK family, including the picture of gene duplications and losses and, perhaps, of unequal evolutionary rates within the family (note many long branches in the GRK4/5/6 and GRK1/7 clades in Fig. 1). Nevertheless, several conclusions about the GRK family evolution can be made with considerable degree of certainty. Most importantly, the RH+KD domain fusion is present in all animals, in two non-metazoan opisthokonts, and in three other lineages of the unicellular eukaryotes. The GRK kinase domain is most closely related to the kinases of the ribosomal protein S6 kinase family ([16] and www.kinase.com), which is ubiquitous in unicellular eukaryotes. Thus, it is most likely that the KD region of GRKs has been produced by duplication of the S6 kinase in the evolution of protists. The provenance of the RH domain is much harder to establish because of the fast evolution of this relatively short region and the uncertain placement of the RH domain of GRKs in the phylogenetic tree of RGS proteins [56], but it is notable that the RH domains are widespread in protists and should have been available for “domain tinkering”.

GRKs recognise and selectively bind activated GPCR, which act as allosteric activators. The N-terminus was implicated in kinase activation by active GPCRs. Mutations in this region specifically impaired the phosphorylation of GPCRs, but not other substrates [32], [43]. The N-terminus was proposed to interact with the kinase domain, stabilizing the alignment of the two lobes necessary for activity [32], [43]. The N-terminus was predicted to form α-helix [32], which was observed in the crystal structure of the most closed active-like conformation of any GRK solved thus far [34]. Interestingly, in this structure the helical conformation of GRK6 N-terminus is stabilized by the crystal contacts, which were proposed to mimic GRK interaction with the receptor [34], support the proposed role of the N-terminal α-helix in GPCR-induced GRK activation. Therefore, it is likely to be the first element to acquire GRK-like characteristics. Our analysis demonstrates that N-terminal elements implicated in receptor binding are recognizable in GRKs from protists and are strictly conserved in all other species (Fig. 3). A stretch of positively charged residues between the starting N-terminal α-helix and the first helix of the RH domain characteristic of the GRKa/4/5/6 family was predicted to participate in GRK binding to the membrane and its proper orientation for receptor interaction [34]. The elimination of these charges in GRK5 blocks the biding to PIP_2_, and precludes PIP_2_–dependent increase in receptor phosphorylation [57]. The sequence comprising these positive charges (Fig. 4) is remarkably conserved in all metazoan species from *T. adherens* and cnidarians, and is also present in an elementary form in GRKa from a non-metazoan opisthokont *Capsaspora owczarzaki*. Thus, it is most likely that this structural element evolved at the root of Metazoa to enhance the efficacy of GRK-mediated receptor phosphorylation.

A duplication of the ancestral GRK must have occurred at some point in the evolution before the emergence of Metazoa, to give rise to the lineage that includes GRKa/1/7/4/5/6 and the other, GRKb/2/3 lineage. Since two unicellular opisthokont genomes appear to encode one GRK each, and these GRKs may belong to two different clades, it is possible that GRKa and GRKb emerged in the opisthokont lineage and preceded the advent of multicellularity. Following this duplication, *Monosiga* may have lost GRKa, and *Capsaspora* lost GRKb. It is also possible that one or both of the “missing” genes simply remain un-annotated in the draft genome assemblies. The pre-duplication stage of GRK evolution is also represented by single-copy GRKs of *Phytophtora*, *Albugo* and brown alga.

The GRKa clade later split into GRK1/7 and GRK4/5/6 lineages – most likely in the early chordates, since an apparently ancestral “visual” GRK is present in the urochordate *C. intestinalis*. This explains the lack of GRK1/7 genes in invertebrates. However, no visual GRK is found in amphioxus, possibly due to a secondary gene loss. Further gene duplications produced GRKs 2 and 3 in the GRK2/3 clade, GRKs 4, 5, and 6 in the GRK4/5/6 clade, and GRK1 and 7 in the GRK1/7 clade. It has been proposed that vertebrates evolved through two rounds of whole-genome duplications (the “2R” hypothesis), first at the root of the vertebrate lineage and the second when jawless vertebrates brunched off [58], [59], [60]. The duplication in the GRKa clade, splitting the family into the GRK1/7 and GRK4/5/6 lineages, coincides with the first duplication. Further increase in the number of GRK isoforms is likely a part of the second duplication at the root of jawed vertebrates, since teleost fishes possess full complement of GRK isoforms. Unfortunately, no fully sequenced genomes between amphioxus and teleost fishes are available for comparison. Our BLAST search in a partial genome sequence of a cartilaginous fish elephant shark *(Callorhinchus milii)*, which has recently become available [61], produced no matches. Cartilaginous fishes represent an oldest living group of jawed vertebrates and are important for understanding vertebrate evolution.

A third full-genome duplication is believed to have occurred in teleost fish lineage [62]. The results of this duplication are reflected in the presence of two GRK5 paralogs in zebrafish. One rapidly evolving paralog, GRK5C, implicated in Wnt signalling in zebrafish [63] diverged significantly in the kinase domain and evolved a novel C-terminus lacking membrane recruitment motifs typical for the GRKa/4/5/6 family. A similar fast evolving GRK5 paralog is present in pufferfish. This is consistent with asymmetrically accelerated post-duplication evolution of one of the paralogs [64]. Duplicates for GRK4 and 6 appear to have been lost, and so has GRK2, substituted, possibly, by a GRK3 paralog. Teleost fishes also retained two closely related paralogs of GRK1 and possibly GRK7.

Split of the ancient eukaryotic GRK into opisthokont-specific GRKa and GRKb appears to have been followed very closely by acquisition of two distinct C-terminal extensions, a PH domain in GRKb and alternative membrane-targeting sequences in GRKa. The large partitions in the GRK kinase domain family trees show close correlation with the identity of the C-terminal regions of GRKs that are involved in membrane binding (Fig. 1). All proteins in the GRKb/2/3 clade in the tree, including the basal GRKb from *Monosiga*, have C-terminal PH domains; all GRK1/7 proteins have either farnesylation or geranylgeranylation CAAX motifs; and all GRK4/5/6 proteins have a predicted C-terminal amphipathic helix in one form or another. The shape of the helix with its characteristic arrangement of hydrophobic and positively charged residues specific for each GRK isoform is strictly conserved in vertebrates. In invertebrates lacking multiple GRK isoforms in the GRK4/5/6 clade, such as Chordata species lancelet and sea squirt, nematode *C. elegans*, placozoan *T. adherens*, and opisthokont *C. owczarzaki*, the form of the helix is GRK4-like, suggesting that this might be the ancestral form preceding gene multiplication in this clade. Insect species have their own unique strictly conserved arrangement, similar to that in cnidarian *Nematostella vectensis*. Thus, different arrangements of the lipid binding residues in the helix appeared in evolution of the GRKa/4/5/6, but some later disappeared or were modified. Although the precise mode of membrane interaction of invertebrate GRKa is unknown, distinct conservation of amphipathic helix suggests a mechanism similar to that operating in mammals.

The GRK1/7 clade is characterized by the presence of a C-terminal prenylation CAAX motif mediating membrane attachment of the members of this GRK subfamily [21], [65], [66]. Mammalian rhodopsin kinases (GRK1) possess well-conserved CAAS motif and are farnesylated [65], [66], whereas cone kinases (GRK7) possessing CAAL motif and are geranylgeranylated [21]. The type of the C-terminal motif correlates well with the placement based on KD sequence (Fig. 1), with one exception: *Gallus gallus* rhodopsin kinase robustly placed in the GRK1 group has CGVL geranylgeranylation motif. The basic member of the “visual” group, a GRK from *C. intestinalis*, also has a geranylgeranylation motif CALL. Thus, geranylgeranylation may have been the lipid modification in an ancestral GRK in the “visual” branch that later changed to farnesylation in the GRK1 subfamily.

The G protein-coupled receptor kinases appear to be a more recent and more restricted addition to the GPCR signaling pathways compared to GPCRs themselves, which are found in nearly all eukaryotes [67]. The origin of some of the GRAFS (Glutamate, Rhodopsin, Adhesion, Frizzled, Secretin) GPCR families could be traced to common ancestor of Uniconts and Alveolates at the very root of eukaryote evolution [68]. We have not found GRKs in plant, fungi, or amoebozoa, whereas all these groups possess GPCRs. It is possible that GRKs have not been properly annotated in these genomes or there was a secondary loss of GRKs in all these lineages except Metazoa and related unicellular groups. GRKs point of origin may be closer to the timing of heterotrimeric G proteins and arrestin-like proteins, which are also found in all Metazoa and in many unicellular eukaryotes. The complete system of phosphorylation recognition, which includes GRKs and arrestins that specifically bind phosphorylated receptors [14], must have appeared at least in the ancestral opisthokont lineage that led to Metazoa. Indeed, *M. brevicolus* genome encodes an arrestin-like protein; *C. owczarzaki* genome encodes two, whereas other GRK-containing unicellular Chromalveolate eukaryotes do not appear to encode any. However, GRKs from unicellular oomycetes and brown algae, although quite different from metazoan GRKs, have perfectly recognizable N-terminal receptor-binding motive (Fig. 3), which suggests that these GRKs might be able to bind and phosphorylate GPCRs. The development of the complete GPCR desensitization system that includes both GRK-dependent receptor phosphorylation and arrestin binding to phosphorylated GPCRs may be linked with the expansion of the Rhodopsin GPCR family that also evolved in the common ancestor of Opisthokonts and enjoyed a huge evolutionary success in Metazoan lineage [67], [68], [69]. It is likely that both these molecular inventions were of use to emerging fast-moving multicellular animals that are constantly surveying the environment with sophisticated sensory systems. This lifestyle requires the ability to reset and re-engage the environmental sensors frequently, which calls for the rapid shut-off mechanism. A dedicated system for quick deactivation of GPCR may have been one of the factors profoundly determining the metazoan lifestyle.

## Materials and Methods

The homologs of GRK proteins were collected with the aid of the BLAST family of programs [70]. The psi-blast search with the KD of *Enteroctopus dofleini* GRK annotated as rhodopsin kinase (gi 4519169) was run with default parameters, except that more matches were allowed to be reported (-b and -v parameters both set to 5000). After two iterations, all known and predicted GRKs from the model organisms were extracted together with many proteins containing RH+KD fusion not annotated as GRKs, followed closely, and sometimes interspersed with, ribosomal protein S6 kinases (RPSK), at E-values less than e^−10^. After verifying that all sequences above this threshold were members of different kinase families, we selected the gi numbers of the top 386 sequences that included many RPSKs, extracted these sequences from the NR database and removed all RPSKs. RPSKs were excluded based on the absence of RH domain. Furthermore, RPSKs group together, at the exclusion of GRKs, in single-linkage clustering and in the phylogenetic tree when they are included in the phylogenetic analysis (data not shown). GRK sequences with incomplete KD, shorter alternatively spliced variants, and GRKs from partially sequenced or incompletely annotated genomes (mostly higher vertebrates as well as several insects). The resulting sequences were aligned using the MUSCLE program [71]. As parts of the RH domain were missing in the GRKs predicted from several genome projects, and the C-terminal regions of GRKs are highly variable, only the aligned kinase domains were used to infer phylogenetic trees (314 aligned sites, starting with the site corresponding to the first conserved beta-strand before the phosphate-binding loop – amino acid 193 in human rhodopsin kinase). Sequences with incomplete RH domain and those missing N- or C-termini were included in the analysis as long as they possessed a complete KD. The Bayesian tree was estimated using MrBayes v.3.1.2 [72]. Maximum-likelihood tree was constructed using the PhyML server [73], with JTT substitution model and gamma shape parameter estimated from the data. The input alignment was resampled to obtain 100 bootstrap replicates, the bootstrapped neighbour-joining trees were obtained, and the percentage of support for every partition in the initial tree was determined.

## Supporting Information

Table S1 **GRK sequences used in this study.**
(DOC)
