# Supplementary material for: The Origin and Evolution of G Protein-Coupled Receptor Kinases
Source: PLoS One. 2012 Mar 19;7(3):e33806. doi: 10.1371/journal.pone.0033806 (PMC3307776; doi:10.1371/journal.pone.0033806)
Supplement: Table S1 — GRK sequences used in this study. (DOC) [file pone.0033806.s001.doc]

Table 1. GRK sequences used in this study.

| N | SPECIES NAME | COMMON ANIMAL NAME | gi NUMBER | ANNOTATED IN GENE BANK | SUGGESTED SYSTEMATIC NAME | Amino Acids |
| --- | --- | --- | --- | --- | --- | --- |
|  | **VERTEBRATES** |  |  |  |  |  |
|  | **Mammals** |  |  |  |  |  |
| 1 | Homo sapiens | Human | 223461377 | GRK1 | GRK1 | 563 |
| 2 | Homo sapiens | Human | 148539876 | GRK2 | GRK2 | 689 |
| 3 | Homo sapiens | Human | 148539879 | GRK3 | GRK3 | 688 |
| 4 | Homo sapiens | Human | 992673 | GRK4 alpha splice variant | GRK4α | 578 |
| 5 | Homo sapiens | Human | 4885349 | GRK5 | GRK5 | 590 |
| 6 | Homo sapiens | Human | 51896039 | GRK6 isoform A | GRK6A[[1]](#footnote-2)* | 576 |
| 7 | Homo sapiens | Human | 51896035 | GRK6 isoform B | GRK6B* | 589 |
| 8 | Homo sapiens | Human | 21166359 | GRK7 | GRK7 | 553 |
| 9 | Pan troglodytes | Chimpanzee | 114638767 | GRK2 *predicted* | GRK2 | 669 |
| 10 | Pan troglodytes | Chimpanzee | 114592958 | GRK4 isoform 7 *predicted* | GRK4 | 578 |
| 11 | Pan troglodytes | Chimpanzee | 114603663 | GRK6 *predicted* | GRK6B* | 582 |
| 12 | Pan troglodytes | Chimpanzee | 114589563 | GRK7 *predicted* | GRK7 | 553 |
| 13 | Pongo abelii | Sumatran orangutan | 197101745 | GRK5 | GRK5 | 485 |
| 14 | Pongo abelii | Sumatran orangutan | 297676821 | GRK6-like, isoform 1 *predicted* | GRK6A* | 576 |
| 15 | Pongo abelii | Sumatran orangutan | 297676823 | GRK6-like isoform 2 *predicted* | GRK6B* | 589 |
| 16 | Macaca mulatta | Rhesus monkey | 109105422 | GRK2 *predicted* | GRK2 | 775 |
| 17 | Macaca mulatta | Rhesus monkey | 109093660 | GRK3 *predicted* | GRK3 | 669 |
| 18 | Macaca mulatta | Rhesus monkey | 297301953 | GRK5-like *predicted* | GRK5 | 664 |
| 19 | Macaca mulatta | Rhesus monkey | 109079929 | GRK6 isoform 5 *predicted* | GRK6A* | 576 |
| 20 | Macaca mulatta | Rhesus monkey | 297295834 | GRK6 isoform 5 *predicted* | GRK6B* | 589 |
| 21 | Macaca mulatta | Rhesus monkey | 109049003 | GRK7-like *predicted* | GRK7 | 553 |
| 22 | Callithrix jacchus | White-tufted-ear marmoset | 296221342 | GRK5 *predicted* | GRK5 | 485 |
| 23 | Callithrix jacchus | White-tufted-ear marmoset | 296193484 | GRK6 isoform 1 *predicted* | GRKA* | 576 |
| 24 | Callithrix jacchus | White-tufted-ear marmoset | 296193488 | GRK6 isoform 3 *predicted* | GRK6B* | 589 |
| 25 | Callithrix jacchus | White-tufted-ear marmoset | 296227941 | GRK7 *predicted* | GRK7 | 553 |
| 26 | Bos taurus | Cow | 27806423 | GRK1 | GRK1 | 561 |
| 27 | Bos taurus | Cow | 27807281 | GRK2 | GRK2 | 689 |
| 28 | Bos taurus | Cow | 27806217 | GRK3 | GRK3 | 688 |
| 29 | Bos taurus | Cow | 194668170 | GRK4 | GRK4 | 588 |
| 30 | Bos taurus | Cow | 27805901 | GRK5 | GRK5 | 590 |
| 31 | Bos taurus | Cow | 296485529 | GRK6 isoform 1 | GRK6A | 583 |
| 32 | Bos taurus | Cow | 27805903 | GRK7 | GRK7 | 552 |
| 33 | Equus caballus | Horse | 149759598 | GRK1 *predicted* | GRK1 | 564 |
| 34 | Equus caballus | Horse | 194218518 | GRK2 *predicted* | GRK2 | 682 |
| 35 | Equus caballus | Horse | 194214161 | GRK3 *predicted* | GRK3 | 662 |
| 36 | Equus caballus | Horse | 194209361 | GRK4-like *predicted* | GRK4 | 676 |
| 37 | Equus caballus | Horse | 194205612 | GRK5-like *predicted* | GRK5 | 605 |
| 38 | Equus caballus | Horse | 194219545 | GRK6-like *predicted* | GRK6A | 576 |
| 39 | Equus caballus | Horse | 194221668 | GRK7 *predicted* | GRK7 | 553 |
| 40 | Sus scrofa | Pig | 194042136 | GRK5 *predicted* | GRK5 | 590 |
| 41 | Sus scrofa | Pig | 47522910 | GRK7 | GRK7 | 553 |
| 42 | Ailuropoda melanoleuca | Giant panda | 301781180 | Rhodopsin-kinase-like *predicted* | GRK1 | 597 |
| 43 | Ailuropoda melanoleuca | Giant panda | 301762368 | GRK4-like *predicted* | GRK4 | 562 |
| 44 | Ailuropoda melanoleuca | Giant panda | 301759209 | GRK5-like *predicted* | GRK5 | 798 |
| 45 | Ailuropoda melanoleuca | Giant panda | 301785534 | GRK6-like *predicted* | GRK6B* | 591 |
| 46 | Ailuropoda melanoleuca | Giant panda | 301762964 | GRK7-like *predicted* | GRK7 | 552 |
| 47 | Canis lupus familiaris | Dog | 73982861 | GRK2 isoform 2 *predicted* | GRK2 | 685 |
| 48 | Canis lupus familiaris | Dog | 73995370 | GRK3 *predicted* | GRK3 | 669 |
| 49 | Canis lupus familiaris | Dog | 73951767 | GRK4 *predicted* | GRK4 | 561 |
| 50 | Canis lupus familiaris | Dog | 73951767 | GRK5 *predicted* | GRK5 | 561 |
| 51 | Canis lupus familiaris | Dog | 73990655 | GRK7 *predicted* | GRK7 | 553 |
| 52 | Oryctolagus cuniculus | Rabbit | 291399899 | GRK7 *predicted* | GRK7 | 553 |
| 53 | Spermophilus tridecemlineatus | Thirteen-lined ground squirrel | 47605756 | GRK7 | GRK7 | 548 |
| 54 | Mus musculus | Mouse | 13633680 | GRK1 | GRK1 | 564 |
| 55 | Mus musculus | Mouse | 13398448 | GRK2 | GRK2 | 689 |
| 56 | Mus musculus | Mouse | 78711830 | GRK3 | GRK3 | 688 |
| 57 | Mus musculus | Mouse | 47605577 | GRK4 | GRK4 | 574 |
| 58 | Mus musculus | Mouse | 148669903 | GRK5 | GRK5 | 572 |
| 59 | Mus musculus | Mouse | 163310714 | GRK6 isoform c | GRK6A* | 576 |
| 60 | Mus musculus | Mouse | 84000007 | GRK6 isoform a | GRK6B* | 589 |
| 61 | Rattus norvegicus | Rat | 149057654 | GRK1 | GRK1 | 564 |
| 62 | Rattus norvegicus | Rat | 6978465 | GRK2 | GRK2 | 689 |
| 63 | Rattus norvegicus | Rat | 6978467 | GRK3 | GRK3 | 688 |
| 64 | Rattus norvegicus | Rat | 12621084 | GRK4 | GRK4 | 575 |
| 65 | Rattus norvegicus | Rat | 13540624 | GRK5 | GRK5 | 590 |
| 66 | Rattus norvegicus | Rat | 163310717 | GRK6 isoform c | GRK6A* | 576 |
| 67 | Rattus norvegicus | Rat | 78214361 | GRK6 isoform a | GRK6B* | 589 |
| 68 | Monodelphis domestica | Gray short-tailed opossum | 126337242 | Rhodopsin kinase-like *predicted* | GRK1 | 565 |
| 69 | Monodelphis domestica | Gray short-tailed opossum | 126324522 | GRK3-like *predicted* | GRK3 | 688 |
| 70 | Monodelphis domestica | Gray short-tailed opossum | 126332159 | GRK4 *predicted* | GRK4 | 578 |
| 71 | Monodelphis domestica | Gray short-tailed opossum | 126291608 | Similar to GRK6 *predicted* | GRK6A | 576 |
| 72 | Monodelphis domestica | Gray short-tailed opossum | 126338152 | GRK7-like *predicted* | GRK7 | 553 |
| 73 | Ornithorhynchus anatinus | Platypus | 149635792 | Rhodopsin kinase-like *predicted* | GRK1 | 565 |
| 74 | Ornithorhynchus anatinus | Platypus | 149641013 | GRK4-like, partial *predicted* | GRK4 | 487 |
| 75 | Ornithorhynchus anatinus | Platypus | 149634652 | GRK5-like *predicted* | GRK5 | 604 |
| 76 | Ornithorhynchus anatinus | Platypus | 149431519 | GRK6-like, partial *predicted* | GRK6B* | 728 |
|  | **Birds** |  |  |  |  |  |
| 77 | Gallus gallus | Chicken | 45382701 | Rhodopsin kinase | GRK1 | 593 |
| 78 | Gallus gallus | Chicken | 71897075 | GRK2 | GRK2 | 688 |
| 79 | Gallus gallus | Chicken | 118098625 | GRK3-like *predicted* | GRK3 | 616 |
| 80 | Gallus gallus | Chicken | 57530046 | GRK4 | GRK4 | 569 |
| 81 | Gallus gallus | Chicken | 118093105 | GRK5-like *predicted* | GRK5 | 590 |
| 82 | Gallus gallus | Chicken | 118097365 | GRK6-like *predicted* | GRK6* | 689 |
| 83 | Gallus gallus | Chicken | 118095069 | GRK7-like *predicted* | GRK7 | 551 |
| 84 | Taeniopygia guttata | Zebra finch | 224050546 | GRK2 *predicted* | GRK2 | 688 |
| 85 | Taeniopygia guttata | Zebra finch | 224050188 | GRK4 *predicted* | GRK4 | 603 |
| 86 | Taeniopygia guttata | Zebra finch | 224052948 | GRK5-like *predicted* | GRK5 | 770 |
| 87 | Taeniopygia guttata | Zebra finch | 224060072 | GRK7-like *predicted* | GRK7 | 551 |
|  | **Amphibians** |  |  |  |  |  |
| 88 | Xenopus laevis | African clawed frog | 62825964 | GRK1 | GRK1 | 549 |
| 89 | Xenopus (Silurana) tropicalis | Western clawed frog | 54606877 | GRK4 | GRK4 | 575 |
| 90 | Xenopus (Silurana) tropicalis | Western clawed frog | 62859911 | GRK5 | GRK5 | 594 |
| 91 | Xenopus laevis | African clawed frog | 148223681 | GRK6 | GRK6A* | 575 |
| 92 | Xenopus (Silurana) tropicalis | Western clawed frog | 187608380 | GRK7 | GRK7 | 551 |
|  | **Fishes** |  |  |  |  |  |
| 93 | Danio rerio | Zebrafish | 62132660[[2]](#footnote-3) | GRK1a | GRK1a[[3]](#footnote-4) | 563 |
| 94 | Danio rerio | Zebrafish | 99028945 | GRK1b | GRK1b | 559 |
| 95 | Danio rerio | Zebrafish | 29561781 | Similar to human adrenergic receptor kinase | GRK2/3 | 591 |
| 96 | Danio rerio | Zebrafish | 197927112 | GRK3 | GRK3 | 688 |
| 97 | Danio rerio | Zebrafish | 121582340 | GRK4 | GRK4 | 573 |
| 98 | Danio rerio | Zebrafish | 292617144 | GRK5-like *predicted* | GRK5 | 578 |
| 99 | Danio rerio | Zebrafish | 260600304 | GRK5 | GRK5-C | 532 |
| 100 | Danio rerio | Zebrafish | 125848675 | GRK6-like *predicted* | GRK6A* | 575 |
| 101 | Danio rerio | Zebrafish | 103472125[[4]](#footnote-5) | GRK7a | GRK7a | 549 |
| 102 | Danio rerio | Zebrafish | 74315906[[5]](#footnote-6) | GRK7B | GRK7b | 548 |
| 103 | Cyprinus carpio | Common carp | 12862625[[6]](#footnote-7) | GRK1 | GRK1a | 563 |
| 104 | Cyprinus carpio | Common carp | 83955370 | GRK1B | GRK1b | 559 |
| 105 | Cyprinus carpio | Common carp | 12862627[[7]](#footnote-8) | GRK7 | GRK7a | 549 |
| 106 | Tetraodon nigroviridis | Green pufferfish | 47230559 | Unnamed protein | GRK1a | 566 |
| 107 | Tetraodon nigroviridis | Green pufferfish | 47226920 | Unnamed protein | GRK1a-like[[8]](#footnote-9) | 570 |
| 108 | Tetraodon nigroviridis | Green pufferfish | 47222525 | Unnamed protein | GRK1b | 550 |
| 109 | Tetraodon nigroviridis | Green pufferfish | 47228567 | Unnamed protein | GRK2 | 697 |
| 110 | Tetraodon nigroviridis | Green pufferfish | 47220260 | Unnamed protein | GRK4 | 628 |
| 111 | Tetraodon nigroviridis | Green pufferfish | 47228574 | Unnamed protein | GRK5-C | 615 |
| 112 | Oryzias latipes | Japanese medaka | 3061337 | OlGRK-R | GRK1a | 563 |
| 113 | Oryzias latipes | Japanese medaka | 319996659 | OlGRK-R | GRK1b | 563 |
| 114 | Oryzias latipes | Japanese medaka | 157278062 | OlGRK-C | GRK7a | 557 |
|  | **CHORDATA** |  |  |  |  |  |
| 115 | Branchiostoma floridae | Florida lancelet | 260826634 | Hypothetical protein BRAFLDRAFT_125081 | GRKa | 525 |
| 116 | Branchiostoma floridae | Florida lancelet | 260820890 | Hypothetical protein BRAFLDRAFT_218343, GRK2 | GRKb | 688 |
| 117 | Ciona intestinalis | Sea squirt | 198421805 | GRK4-like *predicted* | GRK1 | 623 |
| 118 | Ciona intestinalis | Sea squirt | 198414978  198414541 | Beta-adrenergic receptor kinase 2-like, partial *predicted* | GRKb | 446  272 |
| 119 | Ciona intestinalis | Sea squirt | 198437979 | GRK5-like *predicted* | GRKa | 595 |
|  | **INVERTEBRATES** |  |  |  |  |  |
|  | **Insects** |  |  |  |  |  |
| 120 | Drosophila melanogaster | Fruit fly | 17136724 | GRK2[[9]](#footnote-10) | GRKa | 714 |
| 121 | Drosophila melanogaster | Fruit fly | 116007484 | GRK1 isoform A | GRKb | 700 |
| 122 | Acyrthosiphon pisum | Pea aphid | 193669324 | GRK2-like *predicted* | GRKa | 589 |
| 123 | Anopheles gambiae str. | Mosquito | 158291484 | AGAP004117-PA | GRKa | 707 |
| 124 | Anopheles gambiae str. | Mosquito | 158300637 | AGAP012026-PA | GRK6 | 605 |
| 125 | Aedes aegypti | Yellow fever mosquito | 157114479 | Beta-adrenergic receptor kinase | GRKb | 580 |
| 126 | Tribolium castaneum | Red flour beetle | 270013096 | Hypothetical protein TcasGA2_TC011652 | GRKa | 534 |
| 127 | Tribolium castaneum | Red flour beetle | 91088973 | GRK1-like *predicted* | GRKb | 639 |
| 128 | Nasonia vitripennis | Jewel wasp | 156537339 | GRK2-like *predicted* | GRKa | 591 |
| 129 | Nasonia vitripennis | Jewel wasp | 156546725 | GRK1-like *predicted* | GRKb | 686 |
| 130 | Apis mellifera | Honey bee | 66525731 | GRK2-like *predicted* | GRKa | 717 |
| 131 | Apis mellifera | Honey bee | 110759182 | GRK1 isoform 1-like *predicted* | GRKb | 690 |
| 132 | Camponotus floridanus | Florida carpenter ant | 307177632 | GRK2 | GRKa | 695 |
| 133 | Harpegnathos saltator | Jerdon's jumping ant | 307203847 | GRK2 | GRKa | 574 |
| 134 | Pediculus humanus corporis | Human body louse | 242005387 | cAMP-dependent protein kinase catalytic subunit | GRKa | 600 |
| 135 | Ixodes scapularis | Black-legged tick | 241998000 | cAMP-dependent protein kinase catalytic subunit | GRKa | 556 |
|  | **Mollusks** |  |  |  |  |  |
| 136 | Enteroctopus dofleini | Giant octopus | 4519169 | Rhodopsin kinase | GRKb | 689 |
| 137 | Homarus americanus | American lobster | 6175630 | GRK2 | GRKb | 690 |
|  | **Worms** |  |  |  |  |  |
| 138 | Caenorhabditis elegans | Round worm; nematode | 17567137 | GRK1 | GRKa | 642 |
| 139 | Caenorhabditis elegans | Round worm; nematode | 32564603 | GRK2 | GRKb | 707 |
| 140 | Schistosoma mansoni | Trematode flatworm | 256083581 | Serine/threonine protein kinase | GRKb | 1052 |
|  | **METAZOA NON-BILATERIA** |  |  |  |  |  |
|  | **Placozoa** |  |  |  |  |  |
| 141 | Trichoplax adhaerens |  | 196009526 | Hypothetical protein TRIADDRAFT_28308 | GRKa |  |
| 142 | Trichoplax adhaerens |  | 196010207 | Hypothetical protein TRIADDRAFT_59012 | GRKb | 653 |
|  | **Cnidaria** |  |  |  |  |  |
| 143 | Nematostella vectensis | Starlet sea anemone | 156402526 | Predicted protein | GRKa | 549 |
| 144 | Nematostella vectensis | Starlet sea anemone | 156366923 | Predicted protein | GRKb | 384 |
| 145 | Hydra magnipapillata | Hydra | 221108606 | GRK5-like *predicted* | GRKa | 584 |
| 146 | Hydra magnipapillata | Hydra | 221121652 | Similar to bARK1 | GRKb | 417 |
|  | **NON-METAZOA** |  |  |  |  |  |
|  | **Opisthokonts** |  |  |  |  |  |
| 147 | Capsaspora owczarzaki |  | 320164877 | GRK4 | GRKa | 561 |
| 148 | Monosiga brevicollis MX1 |  | 167535332 | Hypothetical protein | GRKb | 541 |
|  | **Other groups** |  |  |  |  |  |
| 149 | Phytophthora infestans | Pathogene causing potato blight | 262106969 | Receptor kinase, putative | GRK | 712 |
| 150 | Albugo laibachii | Plant pathogen causing white rust | 325187777 | Receptor kinase putative | GRK | 651 |
| 151 | Ectocarpus siliculosus | Brown algae | 298711493 | GRK2 | GRK | 686 |

1. ^*^ Splicing variants of GRK6 for all species (marked with asterisks) are suggested to be named according to the human nomenclature. [↑](#footnote-ref-2)
2. This sequence is identical to gi 99028949. [↑](#footnote-ref-3)
3. Duplicates of visual GRKs in teleost fish species are labeled according to their similarity to corresponding variants in zebrafish (Danio rerio). [↑](#footnote-ref-4)
4. This sequence is identical to gi 62132664. [↑](#footnote-ref-5)
5. This sequence is identical to gi 169146175 and 90959375. [↑](#footnote-ref-6)
6. This sequence is identical to gi 83955366. [↑](#footnote-ref-7)
7. This sequence is identical to gi 83955368. [↑](#footnote-ref-8)
8. This sequence is closer to gi 47230559 but is not identical due to gaps and inserts in both sequences. [↑](#footnote-ref-9)
9. This sequence is identical to gi2209349 annotated as GPRK2 (714 aa). [↑](#footnote-ref-10)
